# Ganglionated plexi ablation impact on atrial fibrillation mechanisms and outcomes in patients with low scar burden

**DOI:** 10.1093/europace/euaf178

**Published:** 2025-08-25

**Authors:** Shohreh Honarbakhsh, Caroline Roney, Sayed Al-Aidarous, Caterina Vidal Horrach, Pier D Lambiase, Ross J Hunter

**Affiliations:** Queen Mary University of London, Mile End Road, London E1 4NS, UK; Barts Heart Centre, Barts Health NHS Trust, W Smithfield, London EC1A 7BE, UK; Queen Mary University of London, Mile End Road, London E1 4NS, UK; Barts Heart Centre, Barts Health NHS Trust, W Smithfield, London EC1A 7BE, UK; Queen Mary University of London, Mile End Road, London E1 4NS, UK; Barts Heart Centre, Barts Health NHS Trust, W Smithfield, London EC1A 7BE, UK; Barts Heart Centre, Barts Health NHS Trust, W Smithfield, London EC1A 7BE, UK

**Keywords:** Atrial fibrillation, Ganglionated plexi ablation, Spectral analysis, CS electrogram characteristics, AF inducibility

## Abstract

**Aims:**

Persistent atrial fibrillation (AF) ablation success rates remain limited. The aim was to evaluate the impact of ganglionated plexi (GP) ablation on AF mechanisms and outcomes in patients with low scar burden.

**Methods and results:**

Patients undergoing persistent AF ablation were included. Patients that had <30% low voltage zones (LVZs) in the left atrium underwent pulmonary vein isolation and GP ablation. Dominant frequency (DF), coronary sinus (CS) electrogram characteristics, and AF inducibility score were assessed pre- and post-GP ablation. Ganglionated plexi ablation response was determined. One hundred twenty patients were included, of which 84 (70.0%) patients had <30% LVZs and underwent GP ablation. An ablation response was seen in all patients [AF termination (66.7%) and CL slowing of ≥30 ms (33.3%)]. The average DF, CS cycle length variability (CLV), and CS activation pattern stability (APS) pre-GP ablation were predictive of AF termination. Ganglionated plexi ablation resulted in a significant change in the DF (6.3 ± 1.2 Hz pre-GP ablation vs. 5.1 ± 1.0 Hz; *P* < 0.001), CS CLV (40.2 ± 6.5 ms vs. 28.2 ± 6.8%; *P* < 0.001), and CS APS (25.2 ± 5.8% vs. 35.2 ± 6.5%; *P* < 0.001). Atrial fibrillation inducibility was harder post-GP ablation than pre-GP ablation (3.8 ± 1.2 AF inducibility score pre-ablation vs. 1.3 ± 1.8 AF inducibility score post-ablation; *P* < 0.001). Seventy-eight out of the 84 (92.9%) patients were free from AF/atrial tachycardia (AT) off anti-arrhythmic drugs at 12-months.

**Conclusion:**

Ganglionated plexi ablation results in a mechanistic impact in AF with an ablation response, changes in DF, CS electrogram characteristics, and AF inducibility score. Pulmonary vein isolation and GP ablation in patients with minimal LVZs results in a high freedom from AF/AT.

What’s new?

- This is the first study to evaluate pulmonary vein isolation and ganglionated plexi (GP) ablation in persistent atrial fibrillation (AF) in patients with only a low scar burden and has demonstrated a high freedom from AF/atrial tachycardia during 12-month follow-up of 92.9%.
- This is also the first study to evaluate the impact of GP ablation on AF mechanisms and has shown an ablation response in all patients and a significant change in dominant frequency and coronary sinus (CS) electrogram characteristics with GP ablation. Ganglionated plexi ablation also resulted in a higher AF inducibility score, i.e. harder to induce AF post-GP ablation compared to pre-GP ablation.
- Dominant frequency measurements and CS electrogram characteristics were significantly different in those patients that had AF termination vs. those that had cycle length slowing with GP ablation. Both parameters were strongly predictive of AF termination with GP ablation and can thereby be utilized to elicit the likely ablation response with GP ablation.

## Introduction

Despite advances in understanding of atrial fibrillation (AF), mapping, and ablation strategies, the success rate of persistent AF ablation remains limited. Pulmonary vein isolation (PVI) remains the cornerstone in the ablation of persistent AF and has been shown to be non-inferior to additional linear and complex fractionated electrogram ablation.^1^ However, the success rate of a PVI-only ablation remains only ∼50%.^1^ Therefore, an additional ablation strategy to PVI is being widely sought. Substrate modification with scar homogenization has shown to be a promising ablation strategy in addition to PVI in patients with underlying left atrium (LA) scar.^2^ However, no additional ablation strategy beyond PVI was evaluated in patients with the absence of low voltage zones (LVZs) limiting ablation to PVI only with a success rate of ∼60%.^2^ This study thereby focuses on these patients to evaluate an additional ablation strategy in addition to PVI for this patient cohort.

Autonomic modulation plays a pathophysiological role in AF. Animal studies have shown that temporary suppression of ganglionated plexi (GP) reduces AF burden.^3^ Ganglionated plexi stimulation has also been shown to trigger AF in humans.^4^ In patients, with a low scar burden, focal drivers are more frequently mapped,^5,6^ and focal drivers have been proposed to be secondary to automaticity.^7^ Studies have shown that GP stimulation triggers ectopy.^4,8^ We thereby hypothesize that GP also triggers focal driver activity forming the foundation for the role of GP ablation in persistent AF in patients with minimal LVZs. Even though GP ablation has shown conflicting evidence in persistent AF ablation,^9,10^ these studies have not assessed the underlying LA voltage. Therefore, it remains unclear whether the lack of benefit is because of not limiting the ablation strategy to patients with minimal LA scar. In humans, the mechanistic impact of GP ablation on persistent AF mechanisms is unclear. The mechanistic impact of different ablation strategies have been evaluated through assessing the ablation response,^6,11^ changes in spectral analysis parameters,^12,13^ and AF inducibility.^14,15^ Specific coronary sinus (CS) electrogram characteristics have shown to predict AF/AT recurrence during follow-up^16^; therefore, the impact of GP ablation on these parameters was also sought to be evaluated.

The aim of this study was to evaluate the impact of GP ablation on (i) spectral analysis parameters, (ii) CS electrogram characteristics, (iii) electrophysiological ablation response, and (iv) AF inducibility. The aim of this study was also to evaluate the success rate of PVI and GP ablation in patients with persistent AF and a low scar burden in the LA.

## Methods

Patients undergoing catheter ablation for persistent AF (<24 months and no previous AF ablation) were prospectively included. Exclusion criteria were age < 18 years or reversible cause of AF. Patients provided informed consent for their study involvement, which was approved by the UK National Research Ethics Service (22/PR/0961). The study was prospectively registered on https://clinicaltrials.gov (NCT05633303). Procedures were performed under either conscious sedation or general anaesthetic as per the clinician and patient’s preference. Beta-blocker therapy was stopped in all patients 5 days before the procedure. Because of the long half-life of amiodarone, patients who were on amiodarone continued this. The study methodology is summarized in Supplementary material online, *Figure S1*.

### Electrophysiological mapping

#### Scar assessment

Carto (Biosense Webster, CA, USA) was used as the 3D mapping system. Left atrium anatomical and high-density voltage maps were created using the OctaRay mapping catheter (Biosense Webster, CA, USA). A decapolar catheter (Boston Scientific, MA, USA) was positioned in the CS. The Qdot ablation catheter (Biosense Webster, CA, USA) was used for ablation.

All patients had a high-density bipolar voltage (BV) map created in AF. A minimum of 10 000 points were collected with an interpolation distance of 3 mm to ensure adequate coverage. Any points that were >5 mm from the geometry were deemed to not be in contact and filtered. Three voltage zones were defined: very low voltage zones (vLVZs) < 0.2 mV, low voltage zones (LVZs) was defined as <0.5mV. ^17,18^

Following BV map creation in AF, patients were cardioverted to SR. All patients had a repeat BV map created in SR with atrial pacing at pacing intervals (PIs) of 600 and 250 ms. The 600 and 250 ms BV maps were used to identify fixed and functional remodelling. Low voltage zones identified on the BV PI 600 ms maps were deemed as fixed remodelling. Low voltage zones identified on the BV PI 250 ms maps but not seen on the BV PI 600 ms maps were classified as functional remodelling. Through an automated MATLAB algorithm (MathWorks, USA), the percentage of the LA body [excluding the mitral valve annulus and pulmonary veins (PVs)] occupied by LVZs was assessed. If AF reoccurred during mapping in SR with atrial pacing, patients underwent a further cardioversion to restore SR to allow for completion of the mapping. Fixed and functional remodelling were considered as previous work has shown that both play a role in re-entry formation and rotational driver burden in AF.^17^ If LVZs occupied <30% of the LA body as per the BV PI 250 ms maps, patients were assigned to undergo PVI and ablation of GP sites mapped to the LA body. Atrial fibrillation was re-induced using a pre-defined protocol to assess AF inducibility score pre-ablation (see Supplementary material online, *Figure S2*). Patients then underwent high-frequency stimulation (HFS) to map atrioventricular delay (AVD)-GP sites in AF as described below.

#### Ganglionated plexi mapping

Ganglionated plexi sites resulting in AVD in AF (AVD-GPs) were identified. Ganglionated plexi stimulation was performed with HFS at 50 Hz with an output of 100 V through the distal poles of the ablation catheter using a Grass S88 stimulator (Astro-Med, West Warwick, RI).^19^ Continuous GP stimulation was performed for up to 10 s or until asystole was achieved. Sites that resulted in AVD were tagged as AVD-GP sites on the 3D map. Atrioventricular delay was defined as sites where (i) the RR interval extended by 50% during HFS compared to baseline or (ii) asystole occurred. Baseline RR interval was defined as the mean of 10 RR intervals immediately preceding HFS. The RR interval during HFS was defined as the mean RR interval measured across RR intervals from the first R during HFS and the first R following HFS cessation.^19^ A minimum of 50 sites were stimulated in the LA body in AF, intentionally avoiding the PV ostium and ensuring adequate LA body coverage. Atrioventricular delay-GP sites were targeted because our previous work has shown that these sites overlap anatomically with mapped ectopy-triggered (ET) GP sites and AVD-GP sites are more frequently mapped to the LA body and outside of the PVs compared to ET-GP sites.^18^

#### Pulmonary vein isolation and ganglionated plexi ablation

Following GP mapping, patients underwent PVI with bilateral wide area circumferential ablation (WACA) using radiofrequency ablation (Supplemental Methods). A waiting period of 20 min was applied following PVI to ensure the impact on AF mechanisms was not influenced by PVI. Following PVI, all patients underwent 30-s unipolar recordings in the LA body with a minimum of 30 recordings to ensure adequate LA coverage. Unipolar electrogram recordings over 5 min from a deca catheter positioned in the CS were also performed. The unipolar electrograms were obtained by referencing to the Wilson Central Terminal. These recordings were utilized to perform spectral analysis and CS electrogram characteristic assessment as described below. OctaRay was then placed in the LA appendage (LAA) to monitor AF cycle length (CL). Patients then underwent GP ablation with 45 W and AI targets of 450 anteriorly and 350 posteriorly. Ganglionated plexi sites were ablated with cluster lesions. The ablation was performed at sites that demonstrated a positive response with HFS and with further cluster lesions delivered around these sites. If cluster lesions resulted in a minimal gap that would predispose re-entry circuits, the cluster lesions were joined into a line.

A positive ablation response was defined as (i) CL slowing of ≥30 ms and (ii) AF termination to AT or SR.^6,11^ Cycle length was taken as the average of 30 beats of the electrograms off the OctaRay in the LAA. If a positive ablation response was achieved pre-ablation of all mapped GP sites, the remaining GP sites were ablated. In patients in whom AF did not terminate to AT or SR, repeat 30-s recordings were performed in the LA body with a minimum of 30 recordings to ensure adequate LA coverage. Unipolar electrogram recordings over 5 min from a deca catheter positioned in the CS were also performed. These recordings were utilized to perform spectral analysis and CS electrogram characteristic assessment as described below. These were compared to the pre-GP recordings to evaluate changes in these parameters with GP ablation.

#### Spectral analysis assessment

Dominant frequency (DF) maps were created utilizing the unipolar recordings obtained from all the sequential recordings (Supplemental Methods). Dominant frequency values were used to generate DF maps. The average DF in the LA was used to determine if it was predictive of AF termination with GP ablation, and DF values were compared pre-and post-GP ablation in patients who remained in AF post-GP ablation to determine the impact GP ablation had on DF values.

Using an automated custom-written algorithm that was executed in MATLAB, the anatomical location of mapped GP sites that resulted in AF termination was co-registered onto the DF maps to determine if there was a spatial relationship between sites of highest DF and GP sites that resulted in AF termination. A GP site was deemed to co-locate to a highest DF site if they were within a geodesic distance of 3 mm.

#### Coronary sinus electrogram characteristic assessment

The electrogram characteristics evaluated were CS CL variability (CLV) and CS activation pattern stability (APS). These markers were used to allow assessment of the level of CS organization with regard to both CL and activation pattern. Coronary sinus electrogram characteristics were obtained using a customized automated script written in MATLAB^16^ (Supplemental Methods). The CLV was determined by taking the standard deviation (SD) of CLs (see Supplementary material online, *Figure S3*). A smaller CLV therefore denotes less CL variation and greater CS organization. Coronary sinus APS was determined by assessing the CS activation pattern over a 5-min recording using unipolar electrograms (Supplemental Methods). All the different activation patterns were identified, and the proportion each activation pattern occurred during the 5-min recording was determined. The median of these proportions was taken as the CS APS (see Supplementary material online, *Figure S4*).

#### Atrial fibrillation inducibility score

Atrial fibrillation inducibility score was utilized to compare the ability in inducing AF pre- and post-ablation. Following PVI and GP ablation and restoration of SR either with ablation or cardioversion attempts were made to re-induce AF and to assess the AF inducibility score post-ablation. The induction protocol was consistent with that used by other studies^14^ (see Supplementary material online, *Figure S2*). Atrial fibrillation was deemed to be inducible if it lasted ≥1 min. Atrial fibrillation was deemed to be sustained if it lasted ≥5 min.^14,20^ The ability to induce AF and the AF inducibility score post-GP ablation was compared to the ability to induce AF and the AF inducibility score pre-GP ablation. The relationship between the ability to induce AF post-GP ablation and the AF inducibility score post-GP ablation and AF recurrence during follow-up was also evaluated.

#### Successful ganglionated plexi ablation

To ensure successful GP ablation, repeat HFS was performed at GP ablation sites to ensure no response was elicited as per the criteria defined above. If a repeat response was elicited, further consolidating lesions were performed at the GP site.

#### Follow-Up

All patients underwent 12-month follow-up appointments at 3, 6, 9, and 12 months. Patients underwent 48-h Holter monitoring at 6 and 12 months. A proportion of patients also had continuous monitoring with wearable devices. A 3-month ‘blanking period’ was observed, with all medication including anti-arrhythmic drugs continued during this time. Clinical success was defined as freedom from AF/AT lasting >30 s off anti-arrhythmic drugs after the 3-month blanking period after a single procedure, as per consensus recommendations.^21^

### Statistical analysis

Statistical analyses were performed using staistical package for the social sciences (SPSS) (IBM SPSS Statistics, Version 25 IBM Corp, NY, USA) (Supplemental Methods).

## Results

Out of the 120 patients included in the study, 84 (70.0%) patients had <30% of LVZs in the LA body and underwent PVI and GP ablation. Baseline characteristics and procedural data for the 84 patients are demonstrated in *Table 1*. A majority of the procedures were performed under local anaesthetic and sedation (*n* = 57. 67.9%). All patients were in AF at the start of the procedure. No complications occurred.

**Table 1:** Baseline characteristics and procedural data

Baseline characteristics	Cohort *n* = 84
Age yrs. mean ± SD	60.2 ± 9.5
Male *n* (%)	59 (70.2)
Diabetes mellitus *n* (%)	7 (8.3)
Hypertension *n* (%)	19 (22.6)
TIA/CVA *n* (%)	4 (4.8)
Ischaemic heart disease *n* (%)	4 (4.8)
Cardiac surgery *n* (%)	0 (0)
Cardiomyopathy *n* (%)	40 (47.6)
BMI kg/m^2^ *n* (%)	
20–30	52 (61.9)
31–40	30 (35.7)
>40	2 (2.4)
Obstructive sleep apnoea *n* (%)	5 (6.0)
Left ventricular EF ≥ 55% *n* (%)	40 (47.6)
LA size mm *n* (%)	
30–40	30 (35.7)
41–50	43 (51.2)
>50	11 (13.1)
AF duration months, mean ± SD	15.3 ± 5.5
Previous AT ablation *n* (%)	
Cavo-tricuspid isthmus-dependent flutter	2 (2.4)
Current anti-arrhythmic or rate-controlling strategy	
Beta-blockers including sotalol	71 (84.5)
Amiodarone	55 (65.5)
Flecainide	8 (9.5)
Calcium channel blocker	2 (2.4)
Digoxin	1 (1.2)
Current anticoagulation strategy	
Warfarin	1 (1.2)
Direct oral anticoagulants	83 (98.8)
**Procedural data**	
Local anaesthetic and sedation *n* (%)	57 (67.9%)
Procedural time minutes, mean ± SD	156.7 ± 32.9
Total ablation time minutes mean ± SD	17.0 ± 4.1
Total GP ablation time minutes mean ± SD	8.4 ± 3.4
Fluoroscopy time minutes mean ± SD	2.4 ± 0.8
Dose area product cGycm^2^	41.4 ± 21.2

AF, atrial fibrillation; BMI, body mass index; EF, ejection fraction; GP, ganglionated plexi; LA, left atrium; SD, standard deviation; TIA/CVA, transient ischaemic attack/cerebrovascular accident

### Ablation response

**Figure 1:**
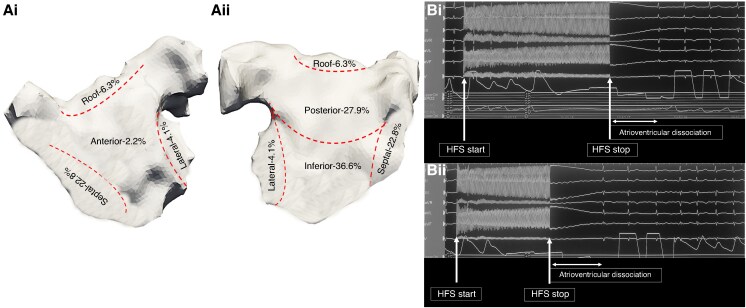
(*Ai*) Left atrium geometry in an anterior–posterior (AP) view demonstrating the distribution of mapped and ablated GP sites using a six-segment anatomical model (anterior, posterior, inferior, lateral, septal, and roof). (*Aii*) Left atrium geometry in a posterior–anterior (PA) view demonstrating the distribution of mapped GP sites using a six-segment anatomical model (anterior, posterior, inferior, lateral, septal, and roof). Most GP sites were mapped and ablated at the posterior and inferior wall. (*Bi–ii*) Atrioventricular dissociation with HFS stimulation in AF at an AVD-GP site. AF, atrial fibrillation; AVD-GP, atrioventricular delay-ganglionated plexi; GP, ganglionated plexi.

**Figure 2:**
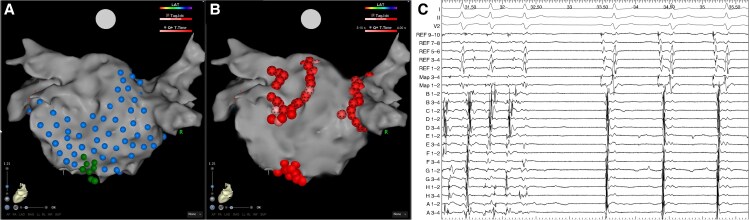
(*A*) Left atrium geometry in a PA view highlighting tagged HFS sites. Blue lesions highlight sites where HFS did not elicit a response that met the criteria for an AVD-GP site. Sites where HFS was performed and resulted in a response that indicated an AVD-GP site are highlighted with a dark green lesion. Ganglionated plexi sites with a response are shown at the low inferior wall. (*B*) Left atrium geometry in a PA view demonstrating ablation of these GP sites, which resulted in SR as shown on the (*C*) electrograms obtained from Bard. The electrograms include the surface ECG and signals from the CS, ablation, and mapping catheter. AVD-GP, atrioventricular delay-ganglionated plexi; CS, coronary sinus; GP, ganglionated plexi; HFS, high-frequency stimulation; PA, posterior–anterior.

**Figure 3:**
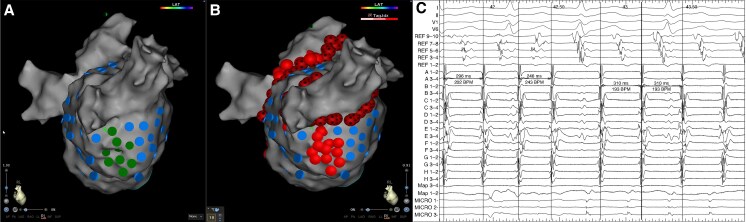
(*A*) Left atrium geometry in a right lateral (RL) view highlighting tagged HFS sites. Blue lesions highlight sites where HFS did not elicit a response that met the criteria for an AVD-GP site. Sites where HFS was performed and resulted in a response that indicated an AVD-GP site are highlighted with a dark green lesion. Ganglionated plexi sites with a response are shown at the septum. (*B*) Left atrium geometry in a RL view demonstrating ablation of these GP sites, which resulted in organization of AF to AT as shown on the (*C*) electrograms obtained from Bard. The electrograms include the surface ECG and signals from the CS, ablation, and mapping catheter. AF, atrial fibrillation; AVD-GP, atrioventricular delay-ganglionated plexi; CS, coronary sinus; GP, ganglionated plexi; HFS, high-frequency stimulation; RL, right lateral.

**Figure 4:**
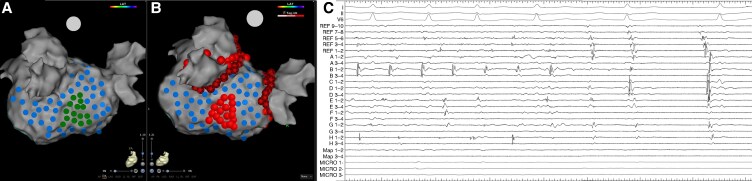
(*A*) Left atrium geometry in a PA view highlighting tagged HFS sites. Blue lesions highlight sites where HFS did not elicit a response that met the criteria for an AVD-GP site. Sites where HFS was performed and resulted in a response that indicated an AVD-GP site are highlighted with a dark green lesion. Ganglionated plexi sites with a response are shown at the posterior–inferior wall. (*B*) Left atrium geometry in a PA view demonstrating ablation of these GP sites, which resulted in initial organization of AF and then termination to SR as shown on the (*C*) electrograms obtained from Bard. The electrograms include the surface ECG and signals from the CS, ablation, and mapping catheter. AF, atrial fibrillation; AVD-GP, atrioventricular delay-ganglionated plexi; CS, coronary sinus; GP, ganglionated plexi; HFS, high-frequency stimulation; PA, posterior–anterior; SR, sinus rhythm.

**Figure 5:**
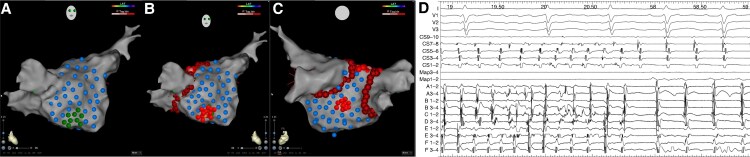
(*A*) Left atrium geometry in a right anterior oblique (RAO) view highlighting tagged HFS sites. Blue lesions highlight sites where HFS did not elicit a response that met the criteria for an AVD-GP site. Sites where HFS was performed and resulted in a response that indicated an AVD-GP site are highlighted with a dark green lesion. Ganglionated plexi sites with a response are shown in the low anterior wall and septum (*B*) Left atrium geometry in a RAO view demonstrating ablation of these GP sites, which resulted in AF cycle length slowing by ≥30 ms. (*C*) A LA geometry in a PA view demonstrating ablation of GP sites with a response on the mid-posterior wall, which resulted in AF organization to an AT as shown on the (*D*) electrograms obtained from Bard. The electrograms include the surface ECG and signals from the CS, ablation, and mapping catheter. AF, atrial fibrillation; AVD-GP, atrioventricular delay-ganglionated plexi; CS, coronary sinus; GP, ganglionated plexi; HFS, high-frequency stimulation; LA, left atrium.

All patients underwent PVI and GP ablation. Following PVI, all patients remained in AF and underwent GP ablation. The mean AF CL pre-GP ablation was 144 ± 5.2 ms and 148 ± 6.8 ms as measured in the CS and LAA, respectively. A total of 268 GP sites were targeted in the 84 patients (3.2 ± 1.5 GP sites per patient). There was no significant difference in the number of GP sites targeted per patient in those that were on amiodarone pre-procedure compared to those that were not (3.1 ± 1.4 on amiodarone vs. 3.3 ± 1.1; *P* = 0.74). There was also no difference in the number of GP sites targeted per patient in those that had their procedure under sedation compared to those that had it under general anaesthesia (3.2 ± 1.2 sedation vs. 3.3 ± 1.2 general anaesthesia; *P* = 0.89). The GP sites were predominantly mapped and ablated on the inferior wall (*n* = 98, 36.6%), posterior wall (*n* = 75, 27.9%), and septum (*n* = 61, 22.8%) (*Figure 1A and B*). The average ablation time for GP ablation was 8.4 ± 3.4 min. Out of the 84 patients, four (4.8%) patients had cluster lesions joined into a line to avoid the risk of re-entry formation. In two patients, this was in the form of a posterior wall line; in one patient, this involved a line from the high septum to the WACA line inferior to the right upper PV; and in one patient, this involved a roof line. An ablation response was achieved in all patients with AF termination being more frequent (*n* = 56, 66.7% AF termination and *n* = 28, 33.3% CL slowing of ≥30 ms). In the 56 patients in whom AF terminated, it terminated to SR in 35 (62.5%) patients and organized to AT in 21 (37.5%) patients (representative *Figure 2A–C*, *Figure 3A–C*, *Figure 4A–C*, and *Figure 5A–C*). In patients that AF terminated to AT, this was mapped to mitral isthmus-dependent flutter (*n* = 8, 38.1%), cavo-tricuspid isthmus (CTI)-dependent flutter (*n* = 14, 66.7%), and roof-dependent flutter (*n* = 3, 14.3%). In four patients, two ATs were mapped, with the first one being mitral isthmus-dependent flutter, which then changed to CTI-dependent flutter on completion of the mitral line. In the 28 patients that had CL slowing of ≥30 ms, all patients underwent unipolar recordings following PVI. All patients had all mapped GP sites ablated. Out of the 56 patients with AF termination, 12 (21.4%) patients had ablation of the remaining mapped GP sites following AF termination. The average additional GP ablation time post-AF termination was 3.1 ± 1.2 min. Atrial fibrillation termination was more frequent when ablating GP sites mapped to the posterior wall (36/75, 48.0%) followed by the inferior wall (13/98, 13.3%). The remaining seven GP sites that resulted in AF termination were mapped to the septum (6/61, 9.8%) and roof (1/17, 5.9%) This was also consistent on a per patient basis, whereby 38 out of 56 patients had AF termination on ablating GP sites mapped to the posterior wall (38/56, 67.9%) followed by the inferior wall (11/56, 19.6%). There was no significant difference in the AF termination rate between the patients who were on amiodarone pre-procedure compared to those who were not (38/55, 69.1% on amiodarone vs. 18/29, 62.1% not on amiodarone; *P* = 0.63). There was also no difference in the AF termination rate between the patients who had their procedure under sedation compared to those who had their procedure under general anaesthesia (38/57, 66.7% sedation vs. 18/27 general anaesthesia, 66.7%; *P* = 1.00).

### Spectral analysis assessment

An average of 42 ± 4.5, 30-s unipolar recordings were performed in the LA body pre-GP ablation in all patients. An average of 45.2 ± 6.3, 30-s unipolar recordings were performed in the LA body post-GP ablation in 28 patients who had CL slowing of ≥30 ms, i.e. remained in AF post-GP ablation. The average DF in the LA body pre-GP ablation was 6.0 ± 1.1 Hz. When comparing the average LA DF pre-GP ablation, in the patients who had AF termination (*n* = 56) vs. the patients who had CL slowing of ≥30 ms (*n* = 28) with GP ablation, the average LA DF was significantly lower in those that had AF termination vs. those that had CL slowing of ≥30 ms (5.6 ± 1.1 Hz, AF termination vs. 6.3 ± 1.2 Hz, CL slowing of ≥30 ms; *P* < 0.001) (*Table 2*).

**Table 2:** Differences in potential predictors for AF termination between the group with AF termination with GP ablation and the group without AF termination with GP ablation

Potential predictors	AF termination on ablation *n* = 56	No AF termination on ablation *n* = 28	*P*-value
CS CLV ms mean ± SD	25.2 ± 5.1	40.2 ± 6.5	<0.001
CS activation pattern stability % mean ± SD	39.2 ± 6.8	25.2 ± 5.8	<0.001
DF Hz mean ± SD	5.6 ± 1.1	6.3 ± 1.2	<0.001
AF duration months mean ± SD	15.4 ± 5.8	14.1 ± 5.4	0.16
LA size cm mean ± SD	43.6 ± 5.1	42.7 ± 5.3	0.22
Amiodarone use *n* (%)	37 (66.1)	18 (64.3)	1.00
Age yrs. mean ± SD	60.3 ± 9.8	60.0 ± 9.1	0.45
Male *n* (%)	39 (69.6)	20 (71.4)	1.00
Cardiomyopathy *n* (%)	26 (46.4)	14 (50.0)	0.82
CVA *n* (%)	3 (5.4)	1 (3.6)	1.00
Hypertension *n* (%)	12 (21.4)	7 (25.0)	0.78
Diabetes *n* (%)	5 (8.9)	2 (7.1)	1.00
Obstructive sleep apnoea *n* (%)	3 (5.4)	2 (7.1)	1.00

AF, atrial fibrillation; CS, cycle length; CLV, cycle length variability; CVA, cerebrovascular accident; DF, dominant frequency; GP, ganglionated plexi; LA, left atrium; SD, standard deviation.

**Figure 6:**
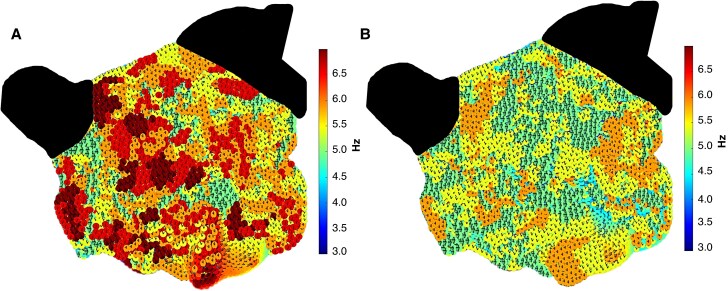
(*A*) Dominant frequency map of the LA in an AP view created in MATLAB using a replica of the geometry created with the 3D mapping system. This demonstrates the DF in the LA body pre-GP ablation. (*B*) Dominant frequency map of the LA in an AP view demonstrated the DF in the LA body post-GP ablation. Comparing these maps, there are more frequent sites with a higher DF on the pre-GP ablation map compared to the post-GP ablation map demonstrating that GP ablation resulted in a reduction in DF measurements. AP, anterior–posterior; DF, dominant frequency; GP, ganglionated plexi; LA, left atrium.

When comparing the average LA DF pre-GP ablation to the average LA DF post-GP ablation in the patients who had CL slowing of ≥30 ms, there was a significant reduction in the average DF with GP ablation (6.3 ± 1.2 Hz pre-GP ablation vs. 5.1 ± 1.0 Hz post-GP ablation; *P* < 0.001) (*Figure 6A and B*). The average LA DF was highly predictive of AF termination with GP ablation with an AUC of 0.84 (95% CI 0.79–0.91; *P* < 0.001). The optimal cutoff for the average LA DF was ≤5.8 Hz, which showed a high sensitivity (83.4%, 95% CI 79.4–92.5%) and specificity (76.8%, 95% CI 73.2–84.3%) in predicting AF termination on ablation. The odds ratio of AF termination on ablation with an average DF of ≤5.8 Hz was 19.2 (95% CI 12.1–26.4; *P* < 0.001).

Out of the 56 GP sites that resulted in AF termination on ablation, 43 (76.8%) co-located to the site of the highest DF. Ganglionated plexi sites that resulted in AF termination were more likely to map to the site of the highest DF compared to GP sites that did not result in AF termination (43/56, 76.8% vs. 10/28, 35.7%; *P* < 0.001).

### Coronary sinus electrogram characteristics assessment

The average CS CLV was 38.2 ± 12.1 ms pre-GP ablation. When comparing the average CS CLV pre-GP ablation, in the patients who had AF termination (*n* = 56) with the patients who had CL slowing of ≥30 ms (*n* = 28), the CS CLV was significantly lower in those patients who had AF termination with GP ablation (25.2 ± 5.1 ms AF termination vs. 40.2 ± 6.5 ms CL slowing ≥30 ms; *P* < 0.001) (*Table 2*). Coronary sinus CLV pre-GP ablation was highly predictive of AF termination with GP ablation with an AUC of 0.86 (95% CI 0.76–0.98; *P* < 0.001). The optimal cutoff for CS CLV was <30 ms, which had a sensitivity and specificity of 86.1% (95% CI 76.2–91.9%) and 83.4% (95% CI 71.2–97.9%) in predicting AF termination on ablation, respectively. The odds ratio of AF termination on ablation in a patient with a CS CLV of <30 ms was 20.1 (95% CI 4.6–35.6; *P* < 0.001). In the patients who had CL slowing of ≥30 ms, there was a significant change in CS CLV with GP ablation (40.2 ± 6.5 ms pre-GP ablation vs. 28.2 ± 6.8% post-GP ablation; *P* < 0.001) (*Figure 4*).

The average CS APS was 34.5 ± 5.4% pre-GP ablation. When comparing the average CS APS pre-GP ablation, in the patients who had AF termination (*n* = 56) with the patients who had CL slowing of ≥30 ms (*n* = 28), the CS APS was significantly lower in those patients who had AF termination with GP ablation (39.2 ± 6.8% AF termination vs. 25.2 ± 5.8% CL slowing ≥30 ms; *P* < 0.001) (*Table 2*). Coronary sinus APS was highly predictive of AF termination with ablation with an AUC of 0.87 (95% CI 0.81–0.95; *P* < 0.001). The optimal cutoff for CS APS was ≥30%, which showed a high sensitivity (84.3%, 95% CI 78.3–89.9%) and specificity (78.4 (95% CI 76.2–85.3%) in predicting AF termination on ablation (*Table 2*). The odds ratio of AF termination on ablation with a CS APS of ≥30% was 24.1 (95% CI 8.2–33.4; *P* < 0.001). In the patients who had CL slowing of ≥30 ms, there was a significant change in CS APS with GP ablation (25.2 ± 5.8% pre-GP ablation vs. 35.2 ± 6.5% post-GP ablation; *P* < 0.001).

### Atrial fibrillation inducibility score

Pre-GP ablation, in all 84 patients, AF was inducible and sustained with an average AF inducibility score of 1.3 ± 1.8. The majority of patients had AF induced with mild stimulation, being allocated a score of 1 (56/84, 66.7%). Post-GP ablation, all patients had attempts to re-induce AF. Out of the 84 patients, AF was not inducible in 50 (59.5%) patients, giving them an AF inducibility score of 4. There was no significant difference in the proportion of patients with non-inducible AF when comparing those that had the procedure under sedation compared to those that had the procedure under general anaesthesia (34/57, 59.6% sedation vs. 16/27, 59.3% general anaesthesia; *P* = 1.00). The average AF inducibility score post-ablation was 3.8 ± 1.2, which was significantly higher than the AF inducibility score pre-ablation (3.8 ± 1.2 pre-ablation vs. 1.3 ± 1.8 post-ablation; *P* < 0.001). In the 34 patients in whom AF was inducible post-GP ablation, it was sustained in 22 (64.7%) patients. Inducible AF (84/84, 100% pre-GP ablation vs. 34/84, 40.5%; *P* < 0.001) and sustained AF (84/84, 100% pre-GP ablation vs. 22/84, 26.2%; *P* < 0.001) was significantly less common post-GP ablation compared to pre-GP ablation.

### Successful ganglionated plexi ablation

All patients demonstrated successful GP ablation with no pre-defined response demonstrated with HFS at the ablated GP sites.

### Follow-Up

All patients underwent Holter monitoring, and 80 patients had continuous monitoring with wearable devices. All 84 patients demonstrated a significant reduction in HR variability when comparing 6-month Holter monitor findings to pre-procedure Holter monitor findings (*P* < 0.001) and when comparing 12-month Holter monitor findings to pre-procedure Holter monitor findings (*P* < 0.001). Out of the 84 patients, 78 (92.9%) patients were free from AF/AT at 12-month follow-up off anti-arrhythmic drugs. Out of the six patients who had recurrence, five had persistent AF and one had paroxysmal AF (see Supplementary material online, *Figure S5*). Out of the six patients who had recurrence of AF, a majority had recurrence at just after the 3-month blanking period (5/6, 83.3%) with an average time to recurrence of 4.1 ± 1.5 months. Five out the six patients underwent a further AF ablation, whilst one patient had rate control with medication. Patients who had AF termination with GP ablation were more likely to have freedom from AF/AT during follow-up compared to patients who did not have AF termination with GP ablation (56/56, 100% vs. 22/28; 78.6%; *P* < 0.001). There was no significant difference in AF/AT recurrence rate between the patients who were on amiodarone pre-procedure and during the blanking period compared to those who were not (4/55, 7.3% on amiodarone vs. 2/29, 6.9% not on amiodarone; *P* = 1.00). There was also no significant difference in the AF/AT recurrence rate between patients who had their procedure under sedation compared to those who had it under general anaesthesia (4/57, 7.0% sedation vs. 1/27, 3.7% general anaesthesia; *P* = 1.00).

Atrial fibrillation inducibility was shown to significantly correlate with AF recurrence during follow-up, whereby out of the six patients with AF recurrence, all had AF induced with burst atrial pacing, whilst out of the 78 patients without AF recurrence, 28 (35.9%) had AF induced with burst atrial pacing (*P* = 0.003). Sustained AF was also more common in those with AF recurrence compared to those without AF recurrence during follow-up (6/6, 100% AF recurrence vs. 16/78, 20.5% no AF recurrence; *P* < 0.001). In the six patients who had AF recurrence during follow-up, they all had an AF inducibility score of 1. The proportion of patients with an AF inducibility score of 1 post-GP ablation was significantly higher in those with AF recurrence compared to those without (6/6, 100% vs. 1/84, 0.01%; *P* < 0.001).

## Discussion

This is the first study that has evaluated PVI and GP ablation as an ablation strategy for persistent AF in patients with <30% LVZs in the LA body, indicating it is an effective ablation strategy in this patient group. This is also the first study that has shown that GP ablation has an impact on AF mechanisms in humans. This study has demonstrated the following findings:

1. Pulmonary vein isolation and GP ablation in patients with <30% LVZs in the LA body resulted in a high freedom from AF/AT during follow-up of 92.9%.
2. All patients had a positive ablation response with GP ablation with AF termination occurring in >50% of the patients.
3. Dominant frequency measurements and CS electrogram characteristics were significantly different in those patients who had AF termination vs. those who had CL slowing of ≥30 ms with GP ablation. Both parameters were strongly predictive of AF termination with GP ablation and can thereby be utilized to elicit the likely ablation response with GP ablation.
4. Ganglionated plexi ablation resulted in a significant change in spectral analysis parameters and CS electrogram characteristics in the patients who had CL slowing of ≥30 ms.
5. Ganglionated plexi ablation increased the AF inducibility score, whereby it was harder to induce AF post-GP ablation.
6. Atrial fibrillation inducibility and AF inducibility score were significantly different post-GP ablation and correlated with AF recurrence during follow-up.

### Ablation strategy in persistent atrial fibrillation

Several ablation strategies have been evaluated in persistent AF. Pulmonary vein isolation has been shown to be non-inferior to additional linear and CFAE ablation.^1^ Localized driver ablation in addition to PVI has also been evaluated as an alternative ablation strategy in persistent AF; however, this has shown conflicting evidence in persistent AF.^6,22^ The recently published Tailored-AF trial that evaluated the ablation of spatio-temporal dispersion sites plus PVI vs. PVI only did not demonstrate a significant difference in the freedom from any atrial arrhythmia during follow-up.^23^ So far, no ablation strategy has shown to be superior to PVI alone, and thereby PVI remains the cornerstone ablation strategy in persistent AF. A majority of ablation strategies applied in persistent AF have not considered the underlying substrate with regard to the underlying voltage. Therefore, the same ablation strategy has been applied regardless of the presence or absence of LVZs. It remains unclear whether this accounts for the conflicting evidence seen. Substrate modification through homogenizing LVZs has been explored as an ablation strategy in persistent AF and has shown promising results in patients with persistent AF.^2^ However, no additional ablation strategy beyond PVI was evaluated in patients with the absence of LVZs limiting ablation to PVI only with a success rate of ∼60%.^2^ In this study, GP ablation in addition to PVI was evaluated in patients with minimal LVZs and was shown to result in a high freedom from AF/AT during 12-month follow-up. Ganglionated plexi ablation in addition to PVI has predominantly been explored as an ablation strategy in patients with paroxysmal AF with promising results.^24,25^ Less data are available on the role of GP ablation as an ablation strategy in persistent AF. One study evaluated GP ablation only in patients with long-standing persistent AF and showed a success rate of <40%, adding PVI at a second procedure improved success rate but only to ∼60%.^9^ Another study evaluating GP ablation has also shown no improvement in freedom from AF/AT in patients with persistent AF.^26^ In these studies, GP ablation was applied in all patients with persistent AF regardless of the underlying voltage; therefore, it is plausible that the discrepancy between the findings of these studies and ours is because of GP ablation not being adopted in the appropriate cohort of patients with persistent AF. As this was a non-randomized study, it remains unclear whether PVI plus GP ablation is superior to PVI-only ablation in this cohort of patients. However, the patients in this study had an average AF duration of 15 months, and a majority of patients had a moderately dilated LA. Thereby, they had potentially more advanced disease in comparison to the persistent AF cohort included in the STAR AF II trial.^1^ Despite the potentially more advanced disease, the success rate seen with GP ablation in this study was much greater than that reported in the STAR AF II trial for a PVI-only ablation strategy. However, to fully answer the superiority of GP ablation plus PVI vs. PVI only, this would require a randomized controlled trial.

### Role of ganglionated plexi ablation in patients with minimal low voltage zones

Ganglionated plexi play a mechanistic role in persistent AF. Temporary suppression of GP with botulinum toxin in animal models prevents the autonomic remodeling secondary to AF and reduces AF burden.^3^ Ganglionated plexi stimulation has also been shown to trigger AF in humans.^4^ In AF patients, there is abnormally increased GP activity, with an increase in the vagal response during GP stimulation.^27^ As a result, GP ablation has been explored as an ablation strategy in AF. Findings from localized driver studies have shown that whilst re-entry rotational activity has a predilection to LVZs, focal driver activity co-locates to non-LVZs.^5,6^ Thereby, focal driver activity is more commonly mapped in patients with minimal LVZs compared to those with LVZs, where re-entry rotational activity is more common. Thereby, in patients with persistent AF and minimal scar, focal drivers are the predominant driver mechanism, which are proposed to be secondary to automaticity.^7^ Ganglionated plexi trigger ectopy, which initiates AF,^18,28^ and it is hypothesized that GP also triggers focal driver activity forming the foundation for the role of GP ablation in persistent AF in patients with minimal LVZs. Limiting GP ablation plus PVI to patients with minimal LVZs could account for the improved freedom from AF/AT during follow-up as it excludes those patients with LVZs, which exhibit rotational re-entry activity as the major driver for their persistent AF.

### Impact on atrial fibrillation mechanisms

Ablation of GP sites resulted in an ablation response in all patients with AF termination to SR being achieved in most patients. Previous studies have shown that acute AF termination is a predictor of long-term procedural success. Out of 20 studies that assessed the impact of AF termination on outcomes, 17 studies reported a significantly improved outcome in patients with procedural AF termination.^29^ These findings were consistent with this study.

Atrial fibrillation termination was more common with ablation of GP sites mapped to the posterior and inferior walls. These findings potentially suggest that these GP sites are mechanistically more important in AF. Thereby, a hierarchical approach should be adopted to GP ablation, whereby GPs mapped to the posterior and inferior wall should be targeted first. In this study, all mapped GP sites were targeted even if AF termination was achieved. The procedural outcomes thereby demonstrate the impact of ablation of all mapped GP sites. Therefore, it is unclear whether limiting any further GP ablation once AF termination is achieved would have similar procedural outcomes. The AFACART study that evaluated localized driver ablation adopted a similar approach, whereby all mapped drivers were ablated post-achieving AF termination.^22^ However, this approach resulted in extensive ablation that could account for the high rates of AT post-procedure.^22^ This was also the case in the Tailored-AF trial,^23^ in which all spatio-temporal dispersion sites were targeted with an average radiofrequency ablation time of >20 min compared to that in the PVI-only ablation arm. The Tailored-AF trial did not demonstrate a difference in freedom from atrial arrhythmia during follow-up compared to the PVI-only ablation arm which could be as a result of the extensive ablation performed in the tailored arm. In this study, the average ablation duration for GP sites was ∼8 min, and in the 12 patients who had additional GPs ablated post-AF termination, the average additional ablation time was 3 min. The ablation duration is thereby significantly shorter than that reported in the AFACART study and Tailored-AF trial, which could account for the lower rate of AT during follow-up seen in this cohort.

#### Coronary sinus electrogram characteristics

The AF termination group had a lower CS CLV and higher CS APS pre-GP ablation compared to the non-AF termination group. A CS CLV of ≤30 ms and a CS APS of ≥30% pre-GP ablation were strong predictors of AF termination with GP ablation with a high diagnostic accuracy. These findings suggest that CS electrogram characteristics can be utilized to elicit the likely ablation response with GP ablation. This is consistent with the findings of a previous study, which showed that a CS CLV and CS APS were strong independent predictors of AF termination.^16^ In addition, when comparing CS electrogram characteristics pre- and post-GP ablation in patients in whom AF did not terminate with GP ablation, there was a significant reduction in the CS CLV and a significant increase in the CS APS post-GP ablation demonstrating that GP ablation results in CS organization, which has been shown to be an independent predictor of freedom from AF/AT during follow-up.^16^

#### Spectral analysis assessment

Pulmonary vein isolation and linear ablation can reduce global LA DF.^12,13^ This study has shown consistent findings whereby GP ablation resulted in a reduction in the average LA DF post-GP ablation compared to that in pre-GP ablation. This indicates that GP ablation has an impact on AF mechanisms by lowering the global LA DF. The average LA DF was significantly lower in patients who had AF termination as the procedural endpoint compared to those who did not have AF termination with ablation. The average DF was shown to be a strong predictor of AF termination with GP ablation having a high diagnostic accuracy. These findings support that the average LA DF can be used as a potential marker for whether AF termination will be achieved with GP ablation.

Furthermore, GP sites that resulted in AF termination were more likely to be mapped to the highest DF site compared to GP sites that did not result in AF termination. Previous studies have shown that ablation at sites of high DF has resulted in CL prolongation or AF termination.^30^ Localized drivers with a greater temporal stability and recurrence rate are more likely to be mapped to high DF sites, and ablation of these drivers is more frequently associated with AF termination.^6,31^ The relationship between mapped GP sites and DF can be used to stratify GPs that are likely to be more mechanistically important in AF.

#### Atrial fibrillation inducibility score

Non-induced AF post-GP ablation correlated with better procedural outcomes. This is consistent with previous studies.^14,15^ The degree of burst pacing inducing AF was also shown to be decisive in predicting AF recurrence during follow-up. In our study, in the six patients who had AF recurrence during follow-up, all had AF induced with mild stimulation, suggesting that the ease of AF induction also played a role in predicting procedural outcomes.

In this study, GP sites were identified with HFS rather than using presumed anatomical location for GP sites. This is consistent with the approach used in other studies.^32,33^ This approach was utilized because our previous work has shown anatomical variation in the GP site location in the LA body between patients.^18^ A previous study demonstrated that anatomical GP ablation was superior to ablation of GP sites identified through HFS in patients with paroxysmal AF.^34^ However, this study focused on patients with paroxysmal AF rather than persistent AF and included GP sites mapped to around the PVs, whilst this study did not. Anatomical GP ablation has the potential to result in more extensive ablation. This is supported by the findings from a trial comparing PVI plus anatomical GP ablation with PVI only and with anatomical GP ablation only,^35^ which reported an average radiofrequency GP ablation time of 46 min, which is significantly greater than the average GP ablation time of 8 min in this study. The aim of this study was to minimize ablation lesion set by using cluster ablation around identified GP sites, particularly to avoid extensive ablation that can predispose to AT particularly in a healthy atrium but also to not impact the study findings through the effect of linear ablation. In view of this, selective ablation of GP sites identified with HFS was utilized in this study.

A majority of studies have performed HFS with the patient being either under general anaesthesia or deep sedation.^32,33^ In this study, a large proportion of patients had effective GP mapping with HFS under light sedation. There are currently no studies that directly compare the effectiveness of light sedation with general anaesthetic for GP mapping with HFS. Pulsed-field ablation, which also involves delivering high voltage electrical pulses, has predominantly been performed under general anaesthesia; however, conscious sedation protocols have been shown to be feasible.^36,37^ One study has shown that pulsed-field ablation performed under conscious sedation compared to general anaesthesia is associated with a higher rate of AF recurrence.^38^ However, in our study, GP mapping with HFS under light sedation did not result in a significant difference in the proportion of GP sites identified, proportion of patients with non-inducible AF, ablation response achieved, or the recurrence rat of AF/AT during follow-up when compared to GP mapping with HFS under general anaesthesia.

### Limitations

This was a proof-of-concept study that evaluated PVI and GP ablation as an ablation strategy in patients with <30% LVZs in the LA body and potentially indicates this is an effective ablation strategy in this patient group. It provides grounds for a formal randomized controlled trial to evaluate whether PVI and GP ablation is superior to PVI-only ablation.

The patient numbers in this study were limited; however, as this was a mechanistic study, the patient numbers are comparable to those of other such studies.^6,11^

All GPs mapped were ablated even if AF termination was achieved. It remains unclear whether it would be sufficient to not ablate the additional GP sites mapped once AF termination has been achieved. This requires further evaluation.

In this study, patients were defined to have a low scar burden if the proportion of LVZs in the LA body was <30%. The effect of GP ablation using other cutoffs needs to be further evaluated.

## Conclusions

This is the first study to evaluate PVI and GP ablation in patients with persistent AF and <30% LVZs. This ablation strategy was associated with a significantly high rate of freedom from AF/AT during follow-up. Ganglionated plexi ablation impacts AF mechanisms. Ablation of mapped GP sites was associated with an ablation response in all patients, with AF termination being achieved in most patients. Atrial fibrillation termination was more common in those with freedom from AF/AT during follow-up. Ganglionated plexi ablation also impacted on the average DF in the LA, whereby there was a reduction in the average DF post-GP ablation compared to pre-GP ablation. Ganglionated plexi ablation also resulted in CS organization with a reduction in the CS CLV and increase in the CS APS. A lower average DF, lower CS CLV, and higher CS APS were more common in patients with AF termination compared to patients without AF termination, and these markers were strong predictors of AF termination with a high diagnostic accuracy. Ganglionated plexi ablation also reduced AF inducibility, the rate of sustained AF, and AF inducibility score, and these markers were predictive of AF recurrence during follow-up. Pulmonary vein isolation and GP ablation in patients with minimal LVZs is a possible effective ablation strategy and requires further evaluation in randomized controlled trials.

## Supplementary Material

euaf178_Supplementary_Data

## Data Availability

The data underlying this article will be shared at reasonable request to the corresponding author.
