# Supplementary material for: Ganglionated plexi ablation impact on atrial fibrillation mechanisms and outcomes in patients with low scar burden
Source: Europace. 2025 Aug 25;27(9):euaf178. doi: 10.1093/europace/euaf178 (PMC13223759; doi:10.1093/europace/euaf178)
Supplement: euaf178_Supplementary_Data [file euaf178_supplementary_data.zip › Supplemental Figure Legend.docx]

**Supplemental Figure Legend**

***Supplemental Figure 1-*** Flow diagram summarizing the study methodology.

***Supplemental Figure 2-*** Demonstrates the AF inducibility score. The lower the score the easier it is to induce AF. The AF inducibility score ranges between 1-6. A score of 1 indicates the induction of AF with an anterograde drive train with 1 extra whilst a score of 6 indicates the inability to induce AF despite an anterograde drive train with 4 extras and Isoprenaline.

***Supplemental Figure 3-*** Demonstrates a CL histogram obtained at one of the electrodes on the mapping catheter, with percentage of recording made up by each CL on the y-axis and CL on the x-axis. Each bar represents a defined CL. For illustrative purposes, less frequent CLs along the mean were excluded. The dashed line (- -) demonstrates the narrowest range of CLs making up 50% of the cycles (under the taller bell-shaped curve). This corresponds to the dominant CL, which is highlighted as a blue bar. The red dashed lines demonstrate the SD of CLs. CL variability was used as a marker of organization. The CL variability at an electrode was defined as the SD of CLs. A smaller SD of CLs indicated a lower CL variability and thereby greater organization.

***Supplemental Figure 4-*** An illustration to aid in demonstrating how CS activation pattern stability (APS) was determined. The illustration shows atrial activations obtained from the 10 electrodes on the deca catheter within the CS. Six atrial activations are included. The first step in determining the CS APS is to identify the overall leading electrode. During the six atrial activations CS5 (highlighted by a *) is leading the other neighboring CS electrodes for five atrial activations. These atrial activations are highlighted by the red box. For one atrial activation again highlighted by the red box CS 6 (highlighted by a *) is leading the other neighboring CS electrodes Therefore the electrode that is leading the greatest proportion of time during the six atrial activations is CS5. This is therefore deemed the overall leading electrode. Following this, the activation pattern between CS5 and its neighboring four electrodes (CS 7, 6, 4 and 3) is determined. There are three activation patterns identified: CS5-CS4-CS3-CS6-CS7 (green arrows), CS5-CS4-CS3-CS7-CS6 (orange arrows) and for the last atrial activation CS5 is not leading with a CS activation pattern of CS6-CS5-CS4-CS7-CS8 (blue arrows). The first activation pattern occurred for two atrial activations (33%), the second 1 for three atrial activations (50%) and the last 1 for one atrial activation (17%). The median of the proportion of time each activation pattern was present is then taken to represent the CS APS, which in this example is 33%.

***Supplemental Figure 5-*** Flow chart demonstrating the proportion of patients free from AF/AT during follow-up and the number of patients with atrial arrhythmia recurrence.
