# Supplementary material for: Ganglionated plexi ablation impact on atrial fibrillation mechanisms and outcomes in patients with low scar burden
Source: Europace. 2025 Aug 25;27(9):euaf178. doi: 10.1093/europace/euaf178 (PMC13223759; doi:10.1093/europace/euaf178)
Supplement: euaf178_Supplementary_Data [file euaf178_supplementary_data.zip › Supplemental Methods.docx]

**SUPPLEMENTAL METHODS**

PVI was achieved with lesions placed 5-10 mm outside the veno-atrial junction aiming for isolation as ipsilateral PV pairs. The anterior border of the left PVs was ablated on the LA appendage ridge where possible, or on the appendage side of the ridge for cases where this was unstable. Lesions were delivered on the venous side of the appendage ridge only where this was necessary to isolate PVs. Ablation was performed with 90W for 4 seconds aiming for a minimum contact force of 8 grams. If first pass isolation was not achieved with 90W for 4 seconds, further ablation index (AI) guided ablation was performed with 45W and AI targets of 450 anteriorly and 350 posteriorly to achieve PVI.

***Spectral analysis assessment***

To determine DF, following filtering off far-field ventricular signals, a Butterworth 2^nd^ order filter was applied to the unipolar signals. Rectification was then performed to take the absolute value of the signal followed by application of a low pass filter to the signal (20Hz Butterworth 2^nd^ order). Spectral analysis was then performed using a combination of periodogram multiplied by a Hamming window and a Welch periodogram to estimate the power spectral density and DF. Four-second windows were used with 50% overlap between the windows. The DF was then determined for each four-second window and the median of these values was taken as the DF for the 30-second recording. This was performed for all the sequential recordings.

***CS electrogram characteristic assessment***

CS CLV was established by reviewing the CS CLs over a 5-minute recording. Each CL was defined as the interval between two consecutive atrial activations. Ventricular far field signals were filtered. To avoid annotations on noise or fractionated electrograms, signals were filtered using the refractory period. All the CL measurements over the 5-minute recording for each electrode were used to create a histogram of the CLs, with all CLs identified on the x-axis (rounded to the nearest whole millisecond) and the percentage of the recording made up by each CL on the y-axis plotted for each individual electrode, for each 5-minute recording, for each patient. The dominant CL of all the CLs was then identified. The dominant CL was defined as the centre of the narrowest range of CLs in the histogram containing 50% of the cycles. The CLV was determined by taking the standard deviation (SD) of CLs (Supplemental Figure 3). A smaller CLV therefore denotes less CL variation and greater CS organization.

CS APS was determined by assessing the CS activation pattern over a 5-minute recording using unipolar electrograms. The first step was to identify the overall leading electrode. This was achieved by determining for each atrial activation the electrode that was leading the other CS electrodes. The electrode that was then leading the greatest proportion of time considering all the atrial cycles during the 5-minute recording was defined as the overall leading electrode. Once the leading electrode had been identified, the activation pattern of the leading electrode relative to the four neighbouring electrodes was determined. For each atrial activation, the activation time differences were compared between the electrode defined as the leading electrode and its four neighbouring electrodes.

The activation time difference allows the order each neighboring electrode is activated to be determined and thereby the activation pattern for each activation. To identify the activation pattern during cycles when the overall leading electrode is not leading, the activation time ascribed relative to the overall leading electrode was negative indicating that the overall leading electrode is following the comparison electrode during that cycle. All the different activation patterns were identified and the proportion each activation pattern occurred during the 5-minute recording was determined. The median of these proportions was taken as the CS APS (Supplemental Figure 4).

***Statistical analysis***

Statistical analyses were performed using SPSS (IBM SPSS Statistics, Version 25 IBM Corp, NY, USA). Continuous variables are displayed as mean ± standard deviation (SD) or median (interquartile range). Categorical variables are presented as numbers and percentages. Chi-square was used for the comparison of nominal variables. The student t-test, or its non-parametric equivalent, Mann-Whitney U test where appropriate was used for comparison of continuous variables. Receiver Operating Characteristic (ROC) analysis was performed, and Odds Ratio were calculated to determine the diagnostic accuracy of DF and CS electrogram characteristics in predicting AF termination with GP ablation. Area under the curve (AUC) was determined. Sensitivity and specificity were also determined. A p-value of <0.05 was deemed significant. P-value of <0.05 was deemed significant.
