# Supplementary figures and images for: Ganglionated plexi ablation impact on atrial fibrillation mechanisms and outcomes in patients with low scar burden
Source: Europace. 2025 Aug 25;27(9):euaf178. doi: 10.1093/europace/euaf178 (PMC13223759; doi:10.1093/europace/euaf178)

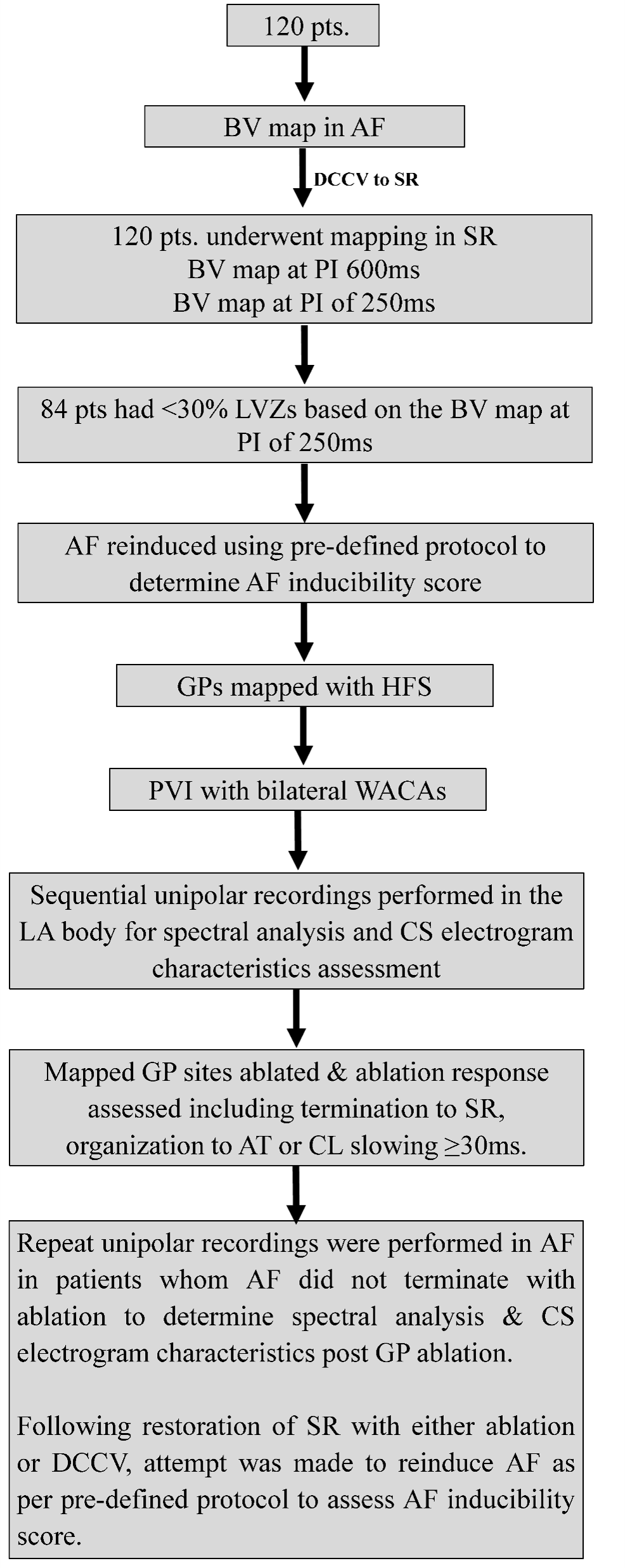

Supplement: euaf178_Supplementary_Data [file euaf178_supplementary_data.zip › Supplemental Figure 1.tif]

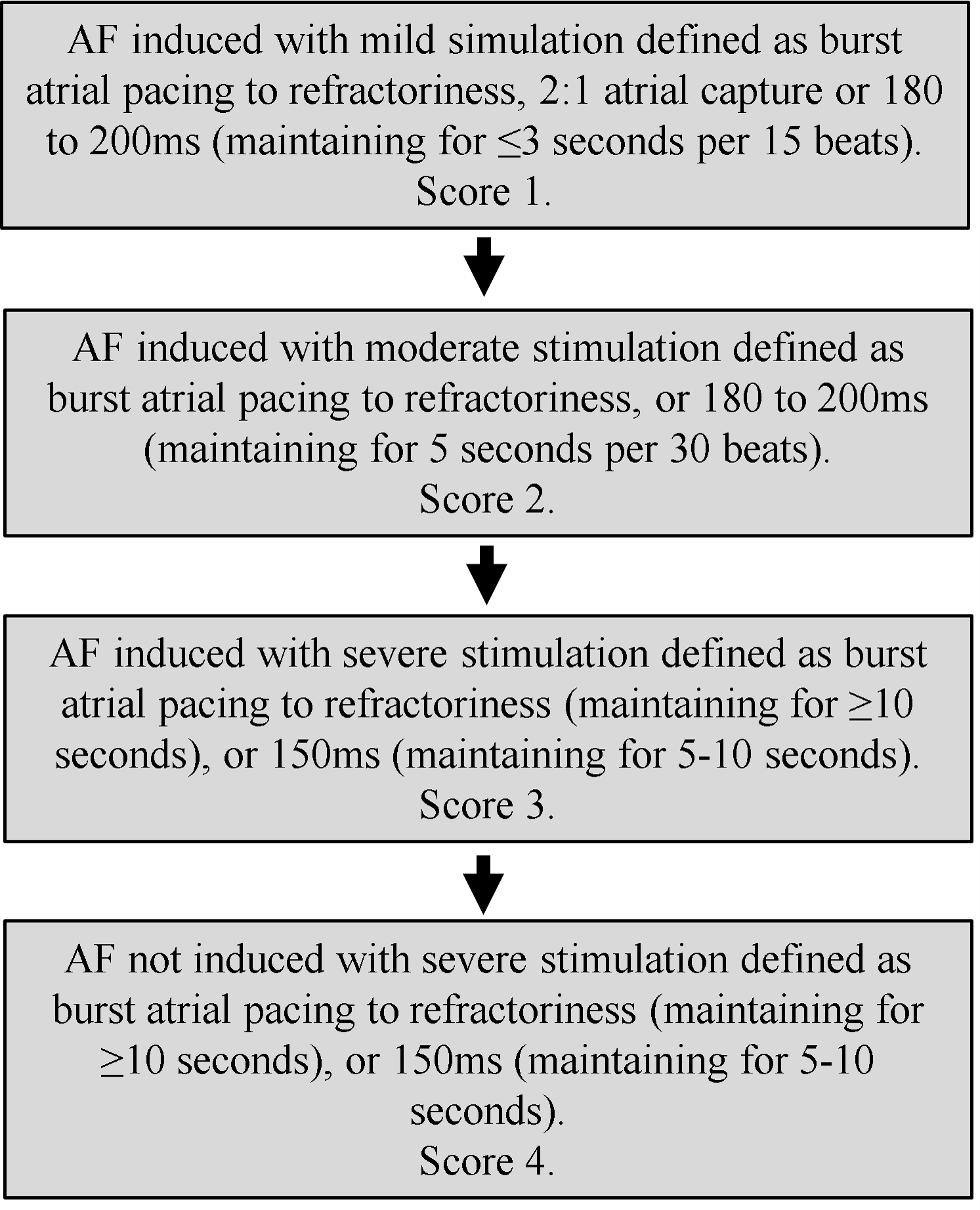

Supplement: euaf178_Supplementary_Data [file euaf178_supplementary_data.zip › Supplemental Figure 2.tif]

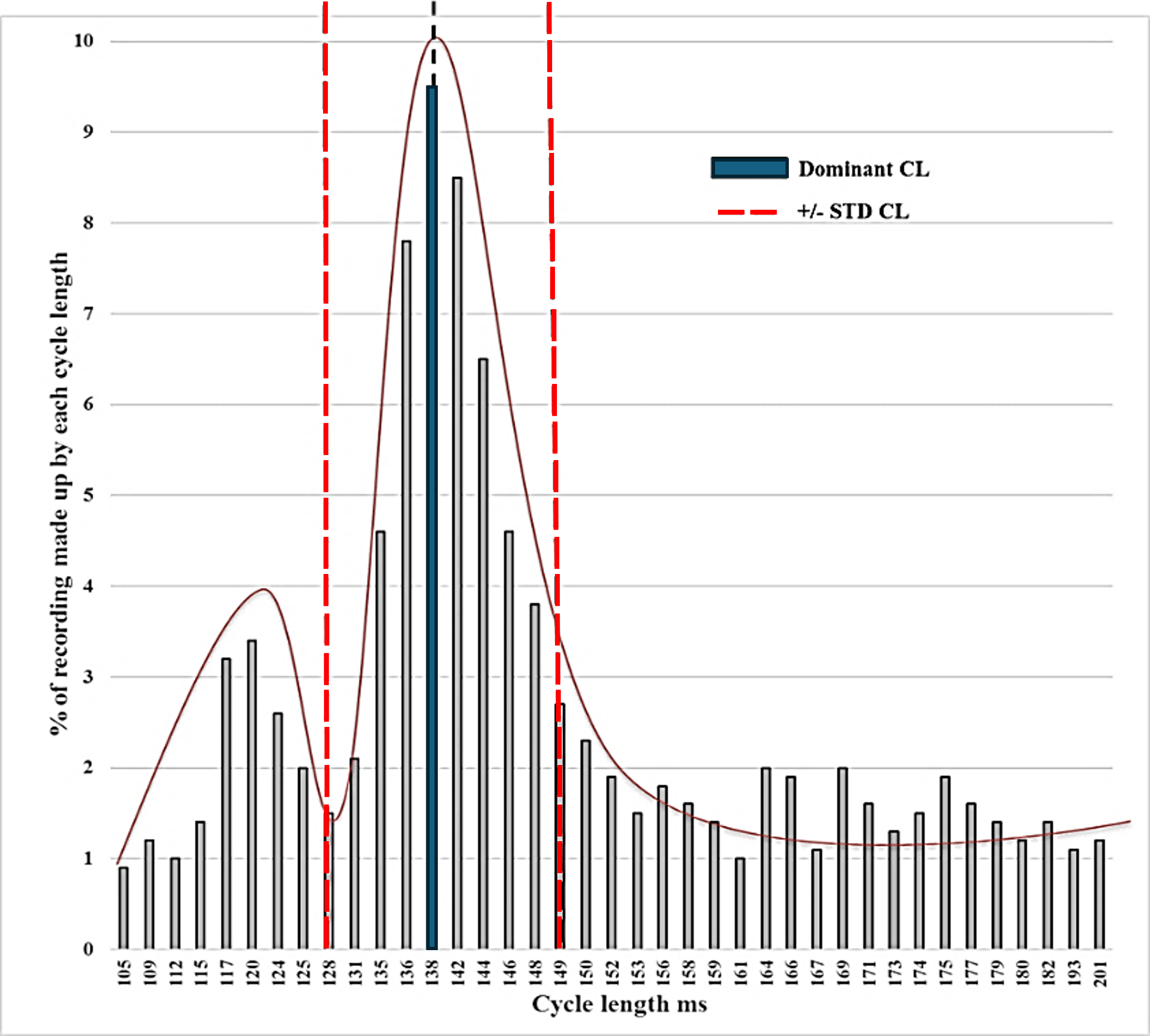

Supplement: euaf178_Supplementary_Data [file euaf178_supplementary_data.zip › Supplemental Figure 3.tif]

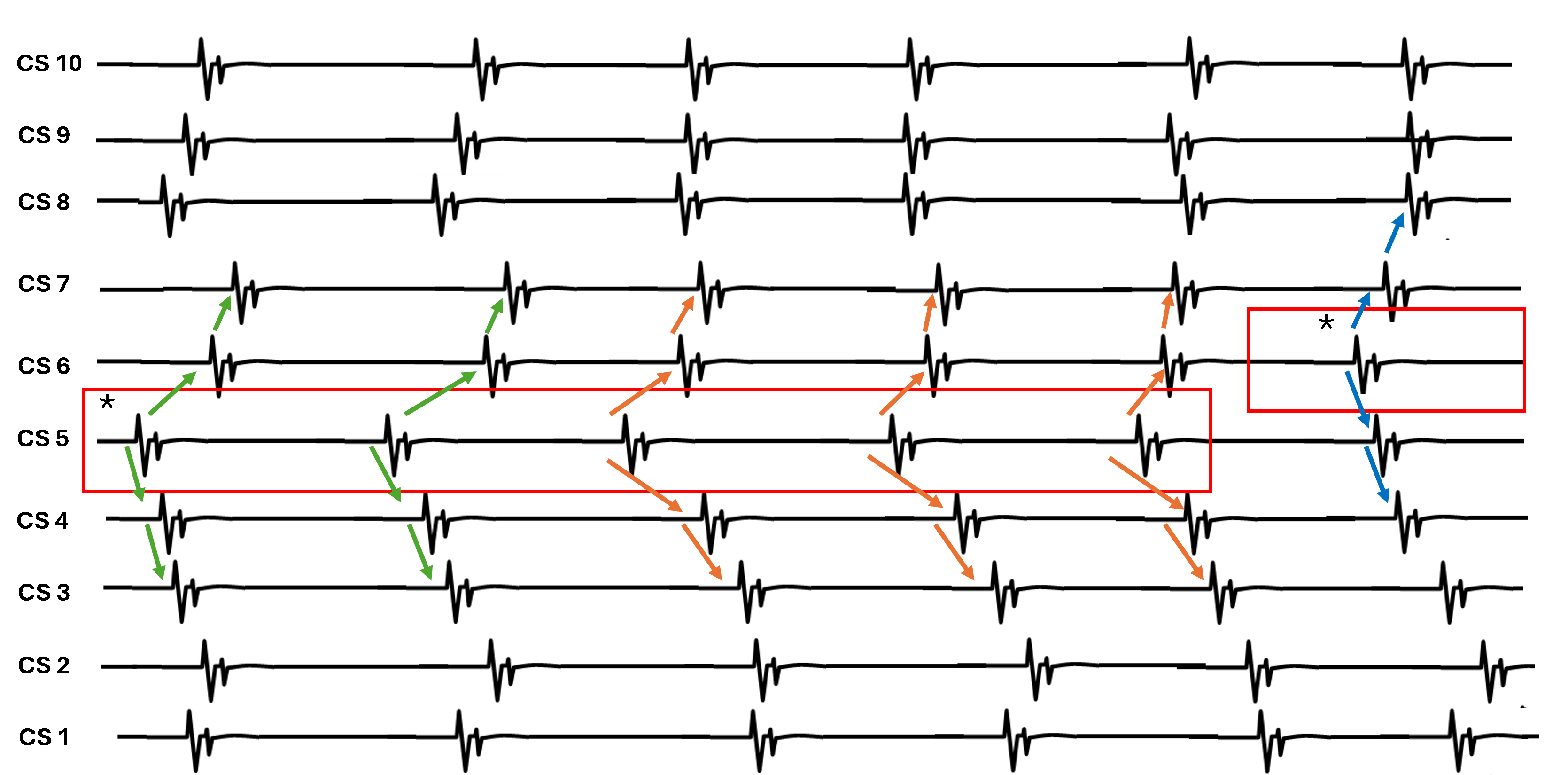

Supplement: euaf178_Supplementary_Data [file euaf178_supplementary_data.zip › Supplemental Figure 4.tif]

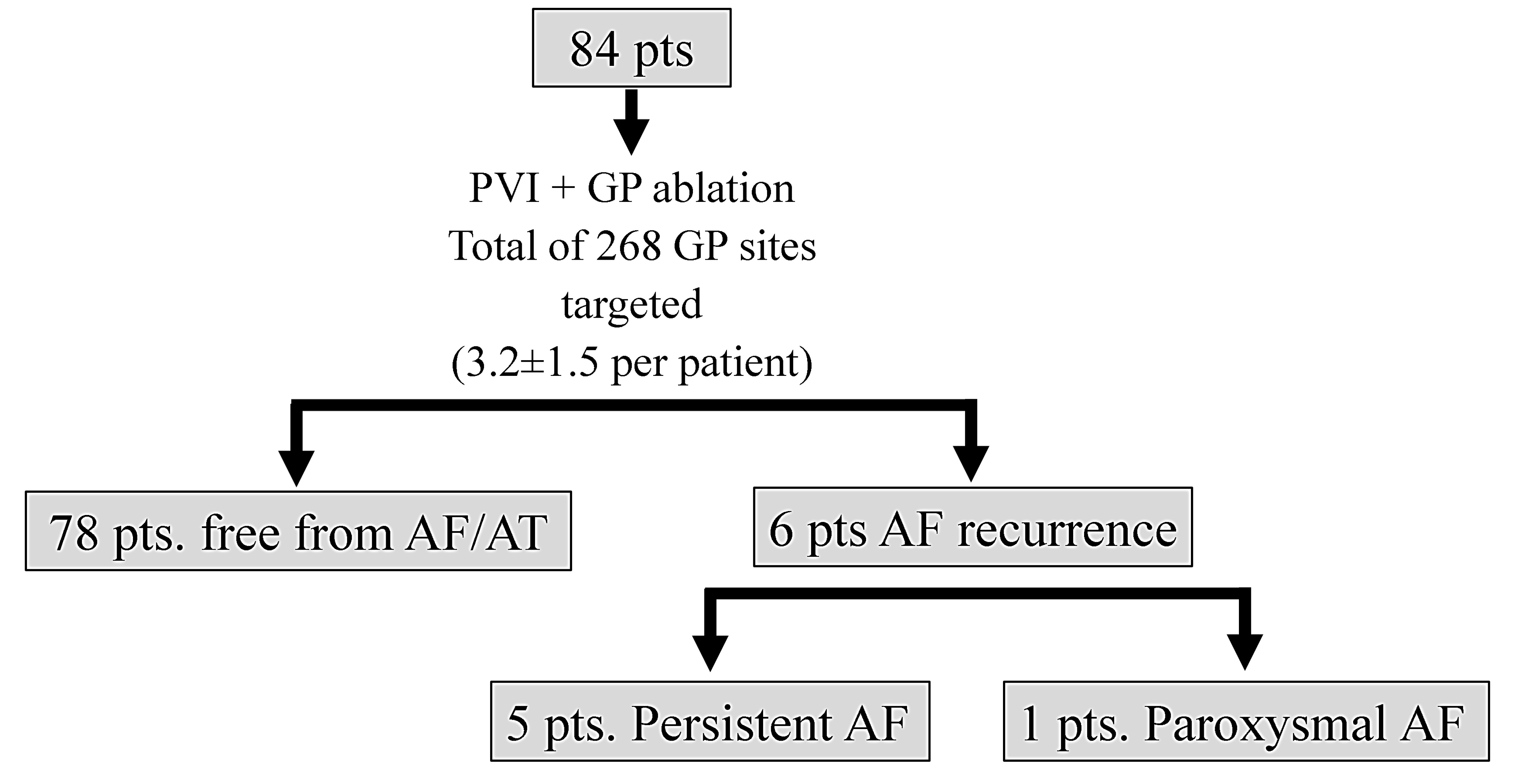

Supplement: euaf178_Supplementary_Data [file euaf178_supplementary_data.zip › Supplemental Figure 5.tif]
